# A new method of alternating shooting of two different seismic sources for deep geophysical surveys

**DOI:** 10.1038/s41598-023-35293-3

**Published:** 2023-06-19

**Authors:** ChenGuang Liu, JinZhong Fu, QingFeng Hua, Kai Liu, PengYao Zhi, YanLiang Pei, QingJie Zhou, XiShuang Li, BaoHua Liu

**Affiliations:** 1 Key Laboratory of Marine Geology and Metallogeny, The First Institute of Oceanography, Ministry of Natural Resources, Qingdao, 266061 China; 2 College of Earth Science and Engineering, Shandong University of Science and Technology, Qingdao, 266590 China; 3 National Deep Sea Center, Ministry of Natural Resources, No. 1 Weiyang Road, Jimo City, Qingdao, 266237 China

**Keywords:** Ocean sciences, Solid Earth sciences

## Abstract

In the latest geophysical survey crossing the Ninety East Ridge of the Indian Ocean, a new method was employed to perform proportional double seismic source excitation and synchronously receive signals from the sea surface and the seabed. The two seismic sources used for excitation were two sets of gun arrays with different energies and dominant frequencies, a G gun array and a Bolt gun array. The G gun array consisted of 3 G.II guns with a total capacity of 450 in^3^ and a dominant frequency of 20–100 Hz. The Bolt gun array consisted of 4 Bolt 1500LL air guns with a total capacity of 6000 in^3^ and a dominant frequency of 10–40 Hz. The seismic receiving system comprised a 480-channel seismic streamer towed from the sea surface and 21 ocean bottom seismometers (OBS). During offshore operations, the integrated navigation system produced equidistant trigger signals at an interval of 50 m. The trigger signals were distributed to the G gun array and Bolt gun array at a ratio of 3:1 after passing through a pulse signal proportional distributor. The two sets of gun arrays fired alternatingly at a given ratio. The receiving equipment on the sea surface and seabed simultaneously received the seismic signals excited by the two sets of gun arrays. After targeted data processing, in addition to the seismic profile generated by the conventional G gun seismic source, the deep seismic profile generated by the Bolt gun seismic source and the survey profile of the active-source OBS were obtained simultaneously. The penetration depths of the three sets of profiles reach 2 km, 6 km, and 30 km, respectively, greatly improving the efficiency of offshore deep-sea seismic surveys.

## Introduction

The marine multichannel seismic (MCS) survey is an important method for detecting mineral resources, the thickness and structure of the sedimentary layer, and fault structures in marine areas. A single air gun or an air gun array is used as the seismic source, and the dominant frequency of air gun seismic sources is generally 20–100 Hz^1–3^. The purpose of an active-source ocean bottom seismometer (OBS) survey is to obtain information about the crustal thickness and deep structures, and a low-frequency and large-capacity air-gun array with a dominant frequency of 10–40 Hz is used as the source^4–7^. MCS and OBS surveys of a geophysical section can be performed simultaneously to obtain information on the morphology, thickness, and structure of shallow sedimentary layers and to provide shallow velocity constraints for OBS inversion, thus improving the inversion accuracy for deep crustal structures.

**Figure 1:**
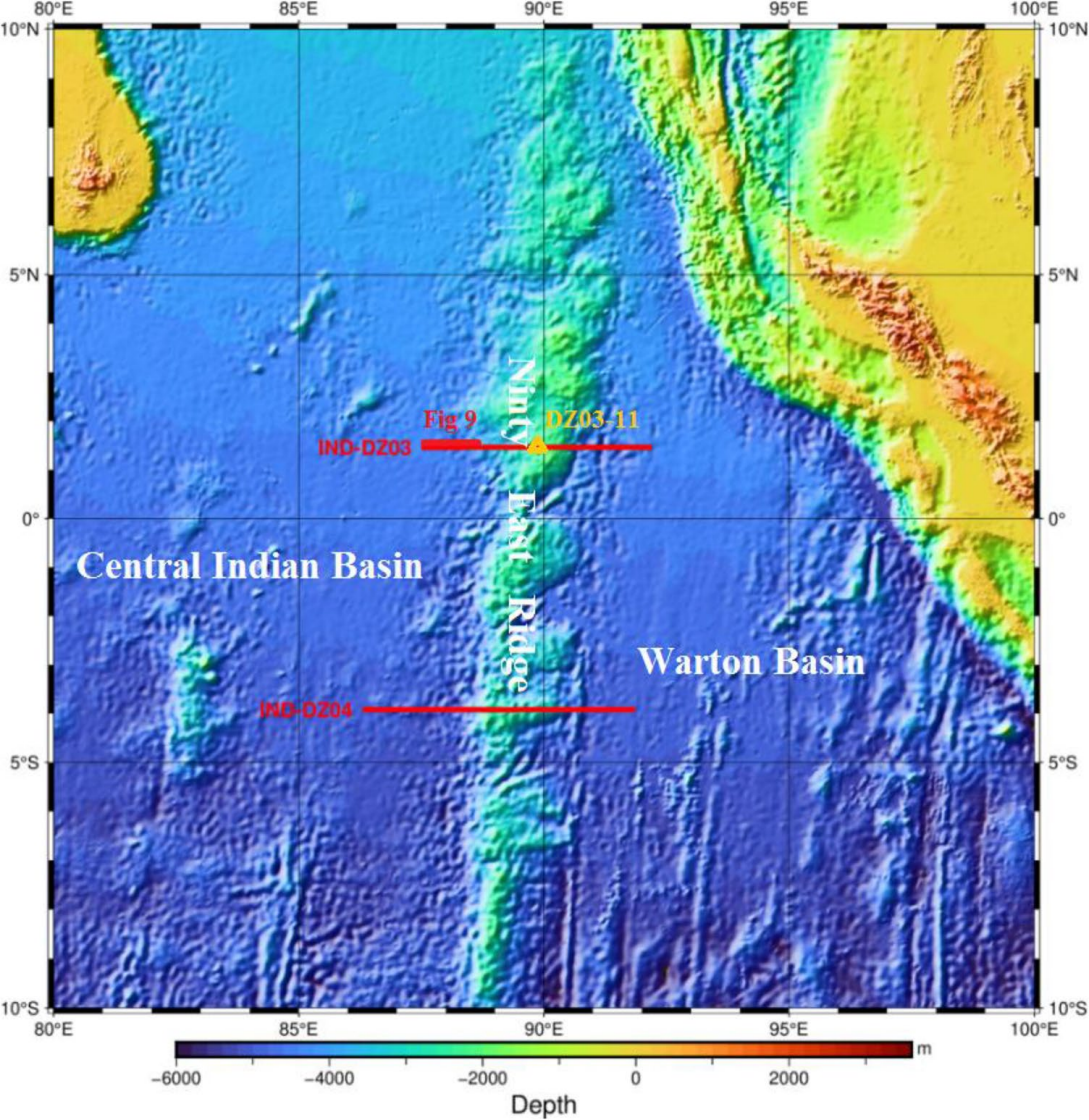
Location of the study area and survey lines. (Drawing software using GMT6.0, grid data from ETOPO1^11,12^).

Due to differences in the survey targets and the dominant frequency of seismic sources, MCS air-gun array firing is commonly performed separately from OBS large-capacity air-gun array firing^8–10^. From January to February 2021, the First Institute of Oceanography, Ministry of Natural Resources, carried out two geophysical survey lines across the Ninety East Ridge of the Indian Ocean with the R/V Xiang Yang Hong 01. In order to save investigation time and improve investigation efficiency. In this survey, both active-source OBS and MCS surveys were conducted, with the total length of the two profiles reaching 1147.5 km (see Fig. 1). During the surveys, the MCS air-gun array and OBS large-capacity air-gun array were fired alternatingly for the first time. After one shot, three profiles with different survey depths and resolutions were obtained, which led to a good survey effect, shortened the voyage of the ship and improved the work efficiency.

## Methods

### Air gun seismic source

**Figure 2:**
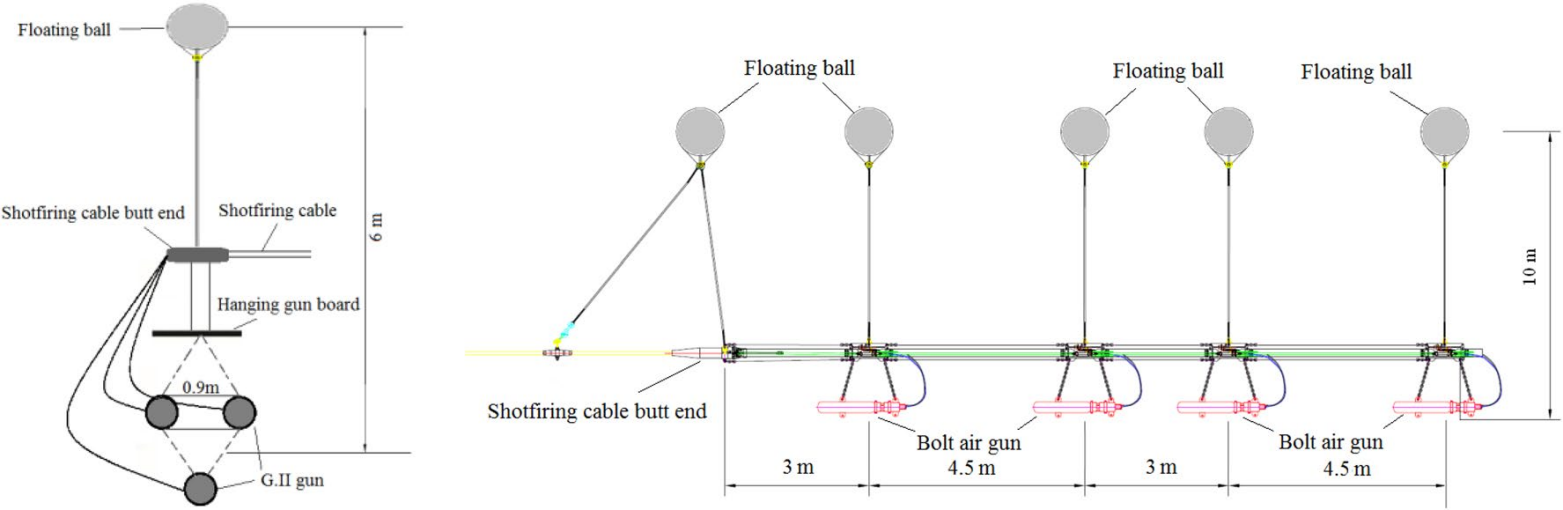
Air gun seismic source arrangement. (The figure on the left is the delta seismic source, and the figure on the right is the Bolt gun seismic source).

In the MCS survey, a G gun array composed of three 150 in^3^ G.II guns was used, with the three air guns arranged in a "Δ" shape, which is also known as a delta seismic source. The distance between the three air guns was 0.9 m, the seismic source was 6 m from the water surface, and the total capacity of the air guns was 450 in^3^. During the active-source OBS survey, a Bolt gun array composed of 4 Bolt 1500LL air guns was used, with a total capacity of 6000 in^3^. The distance between the air guns and the water surface was 10 m. The air gun spacing is shown in Fig. 2.

**Figure 3:**
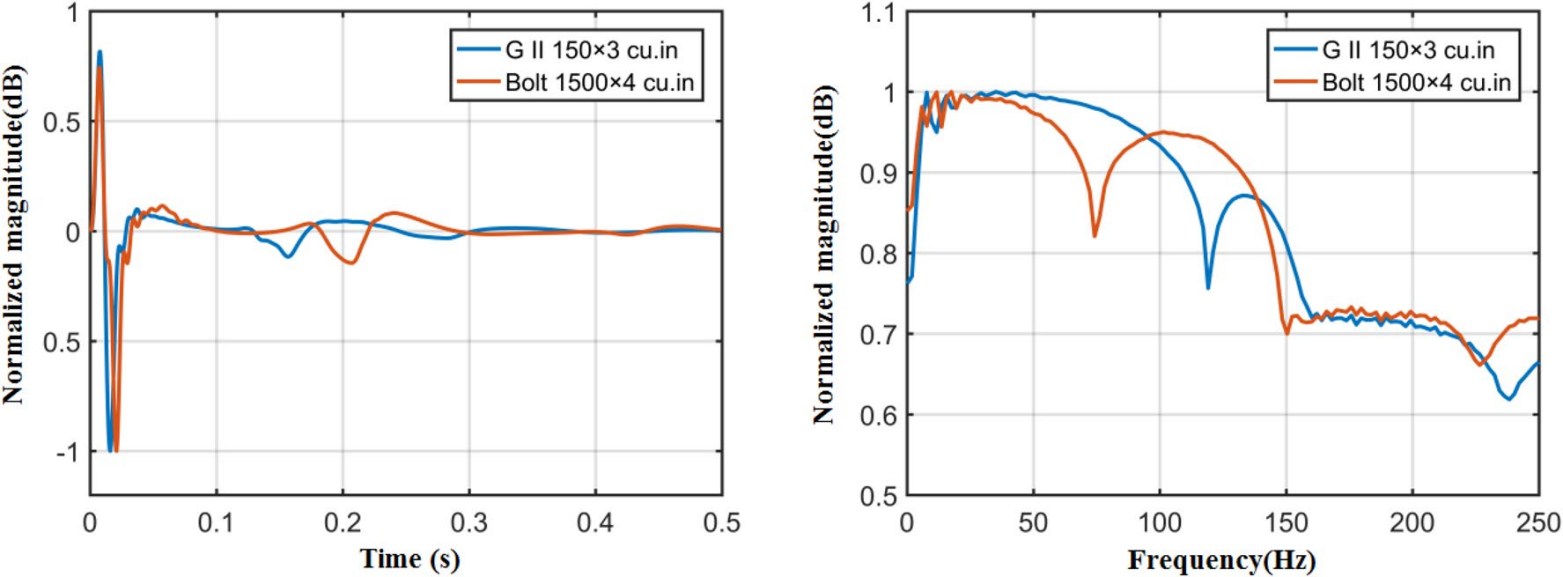
Wavelet simulations of the delta seismic source (450 in^3^) and Bolt gun seismic source (6000 in^3^). (The panel on the left is the simulation results of the far-field wavelet, and the panel on the right is the far-field wavelet spectrum).

The air-gun array wavelet simulation can effectively and theoretically simulate the far-field wavelet morphology and spectrum characteristics of air-gun arraywith different combination methods and provide a theoretical basis for the design and optimization of air gun seismic source parameters during field seismic surveys and acquisition construction^13^. Figure 3 shows the far-field wavelet theory simulation results of the air-gun array used for this voyage. This figure clearly shows that the delta seismic source composed of G.II guns has a high dominant frequency and a wide effective frequency band, which can satisfy the requirements of shallow high-resolution reflection seismic surveys for seismic source wavelets. The Bolt gun is a large-capacity air gun seismic source with a capacity of up to 6000 in^3^. It can excite strong seismic waves with a low dominant frequency and a high energy at the low-frequency end of the far-field wavelet spectrum, which increases the exploration depth of seismic surveys and can yield more information on tectonic structures at depth.

### MCS and OBS signal acquisition

**Figure 4:**
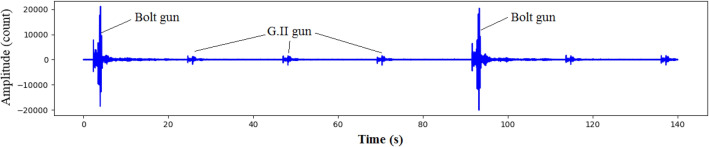
Signals of the two air gun arrays recorded by the MCS system.

The Seal 428 acquisition system was used for MCS acquisition. The total number of channels of seismic cables was 480, and the spacing between channels was 6.25 m. Three types of OBS were employed to receive signals from the gun arrays, including the GeoPro SeidisVI made in Germany, MicrOBS made in France, and PanGui OBS developed by South University of Science and Technology of China. The MCS cables and OBS system simultaneously received signals from the two air gun sources. Figure 4 shows the signals of the two air gun arrays recorded by the MCS system.

### Working process

The first step was to place the OBS cloth on the seabed, followed by laying the MCS cable. The Bolt gun and delta seismic sources were then deployed into the water and fixed to the left and right sides of the stern, respectively. The integrated navigation software SeaproNav triggered the firing of the guns according to a shot spacing of 50 m. The parameters of the seismic profiles are shown in Table 1.

**Table 1:** Parameters of the surveyed profiles.

	G gun seismic profile	Bolt gun seismic profile	Active source OBS profile
Seismic source capacity	450 in^3^	6000 in^3^	6000 in^3^
Shot spacing	50 m	200 m	200 m
Channel spacing	6.25 m	6.25 m	20 km
Total number of channels	480 channels	480 channels	21 stations
Fold number	30	7	–

**Figure 5:**
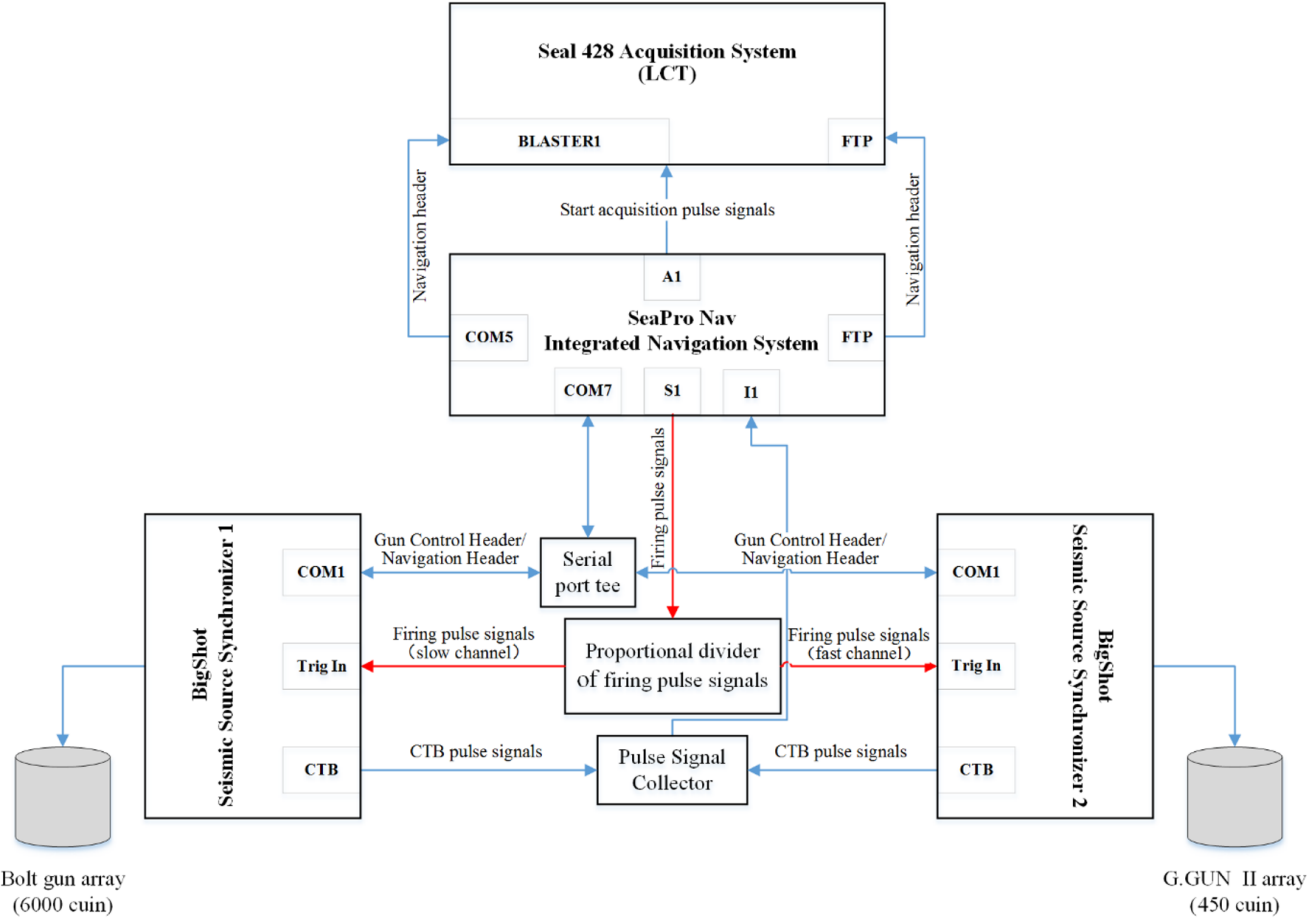
Seismic source excitation system.

A signal distributor was specially designed to send the received trigger signals to two Bigshot air gun controllers at a ratio of 3:1, and then the two air gun controllers excited the delta seismic source and the Bolt gun seismic source (Fig. 5).

## Data processing

### MCS data processing

**Figure 6:**
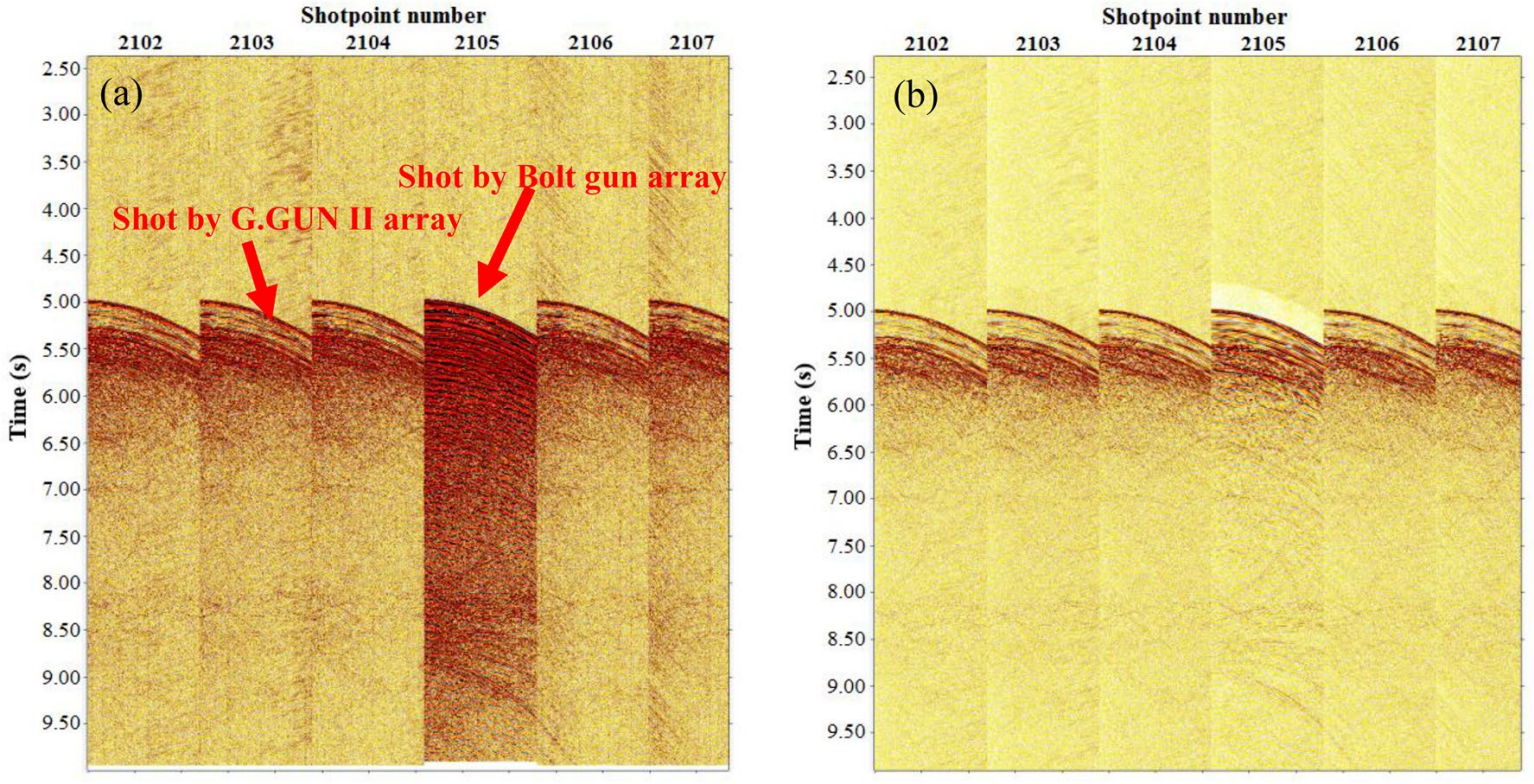
Comparison of the energy equilibrium effects of different shots. ((**a**) Before equilibrium, (**b**) after equilibrium).

**Figure 7:**
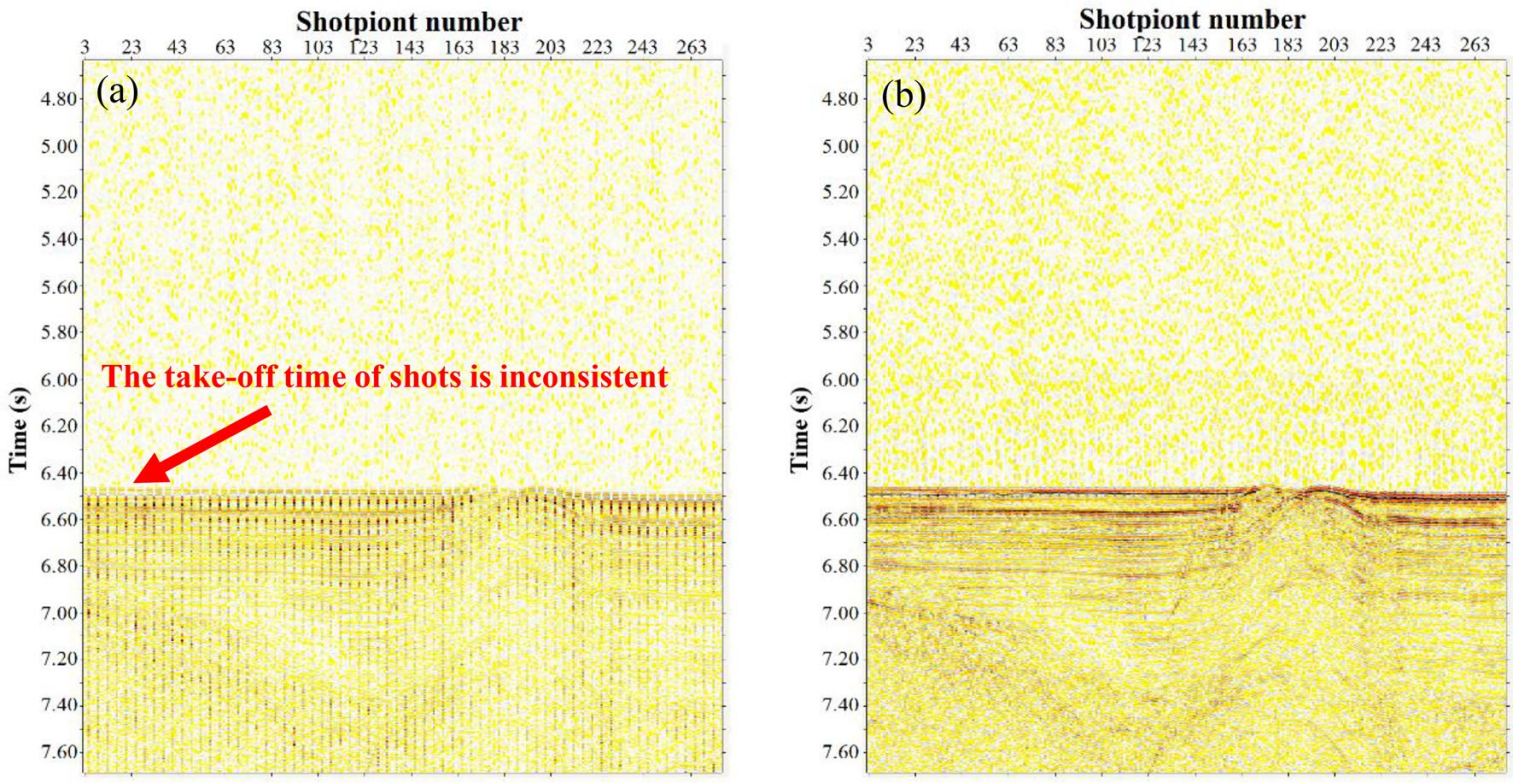
Comparison of the correction effect for the take-off time of different shots. ((**a**) Before correction, (**b**) after correction).

**Figure 8:**
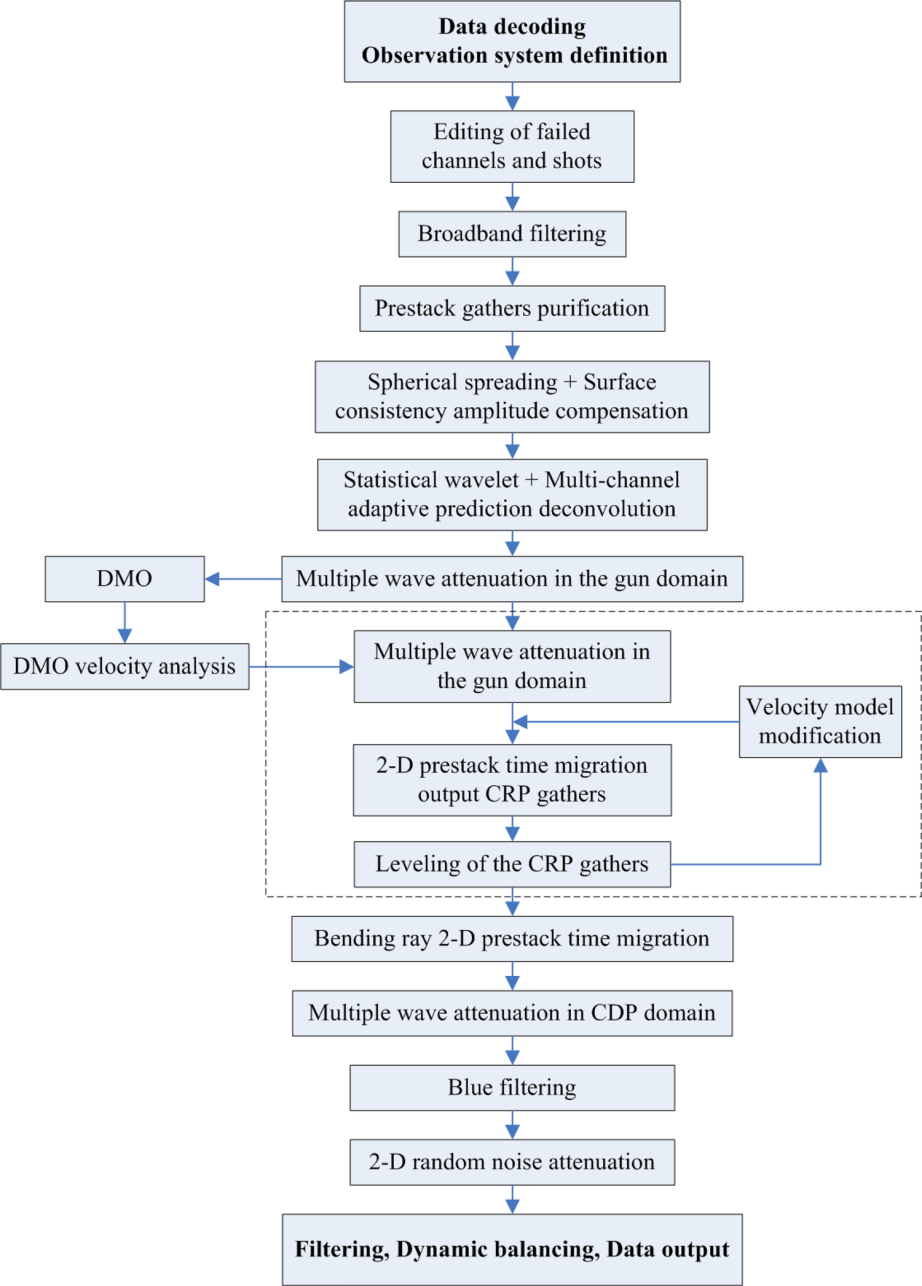
MCS data processing flow.

Compared with routine MCS data, due to the use of two completely different AIR GUN ARRAY seismic sources firing alternatingly, the original seismic shot data set had problems, such as an uneven energy and inconsistent waveform take-off times for different shot energies, as shown in Figs. 6 and 7. These problems had to be suppressed in the pretreatment stage. In addition, the very-low-frequency interference on the seismic data of this voyage was relatively well developed, and the dominant energy was mostly approximately 1–4 Hz, which was easy to suppress while processing. The prevailing lateral reflection waves had a greater impact on the offset and needed to be suppressed. The air gun seismic source wavelet produced an evident bubble effect, which interfered with shallow strata imaging and easily formed a structural illusion. Multiples of the seabed were relatively well developed, which was also the main factor affecting the signal-to-noise ratio of the data in this area. The above types of interference waves require targeted processing. Figure 8 shows the processing flows of the MCS data. The key processing techniques included prestack gather purification processing, combined deconvolution processing^14^, multidomain combined multiple attenuation processing^15,16^, and curved ray Kirchhoff prestack time migration imaging^17^.

**Figure 9:**
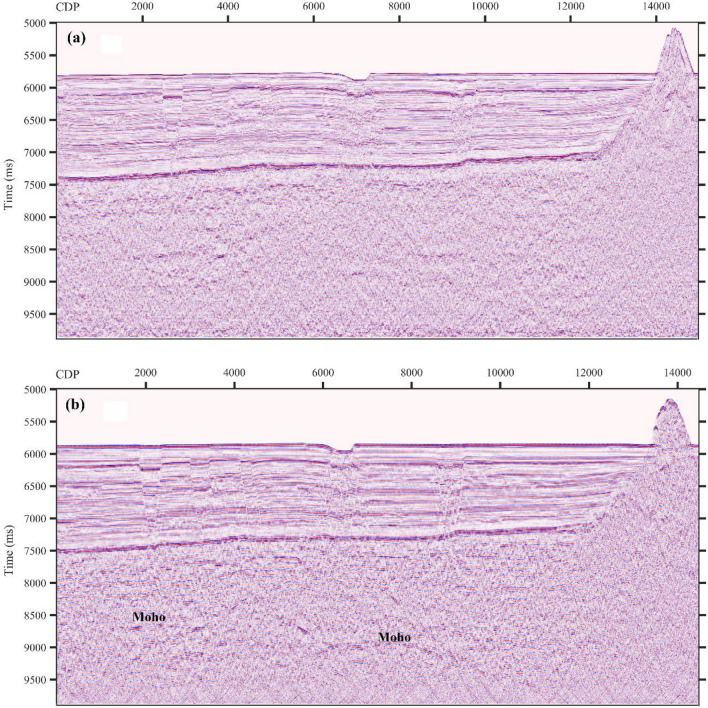
Processing results of the DZ03 line (local). ((**a**) The seismic profile by the G.II gun; (**b**) the seismic profile by the Bolt gun).

**Figure 10:**
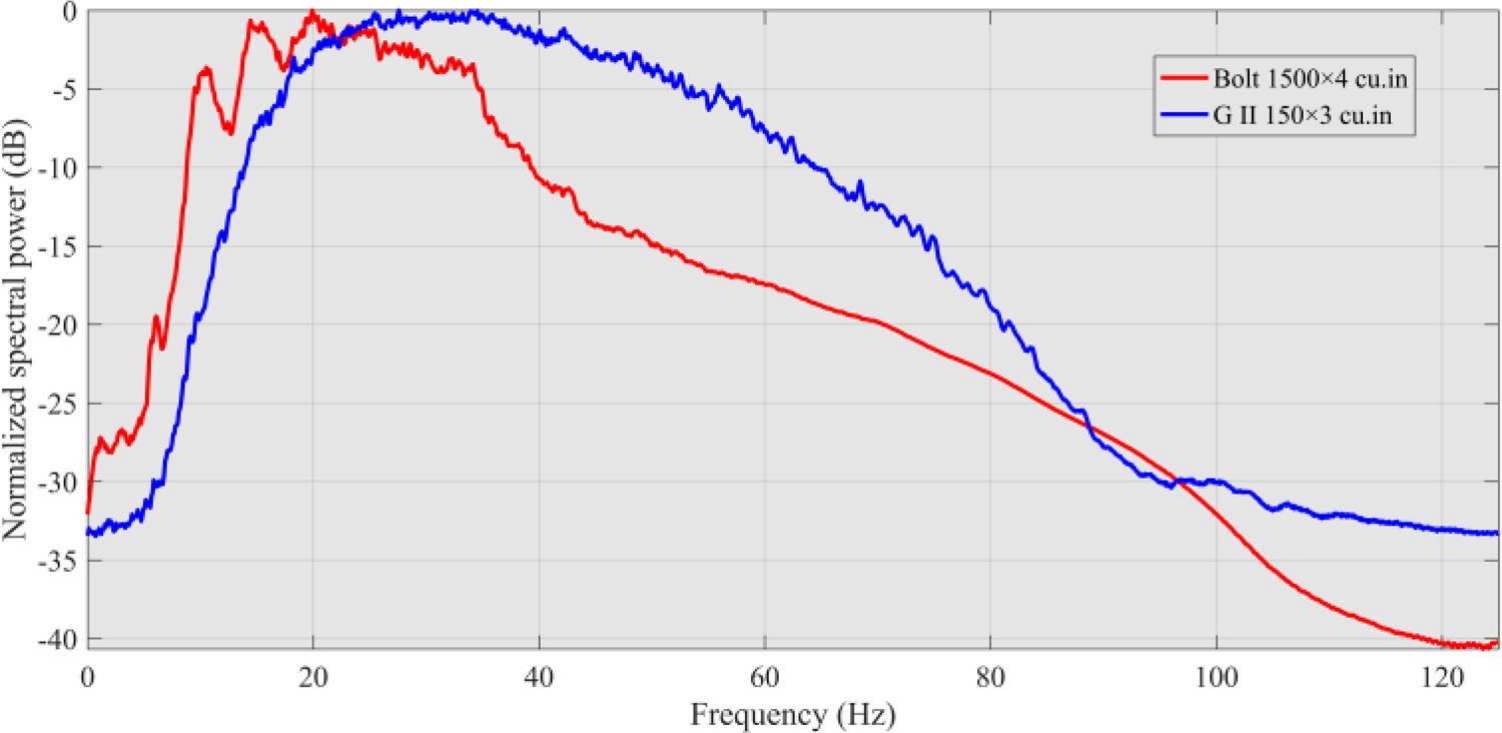
Imaging profile spectrum of the seismic energy produced by alternatingly exciting two different seismic sources.

Finally, according to the original MCS data collected by different alternatingly excited seismic sources, two sets of seismic imaging profiles were obtained. Figures 9 and 10 show two different seismic profiles and spectrum analysis results. The seismic profile obtained by the G gun source has a higher frequency band and a higher resolution (see Fig. 9a), while the seismic profile obtained by the Bolt gun source has a deeper detection depth and can receive reflected signals from the Moho (see Fig. 9b).

### OBS data processing

**Figure 11:**
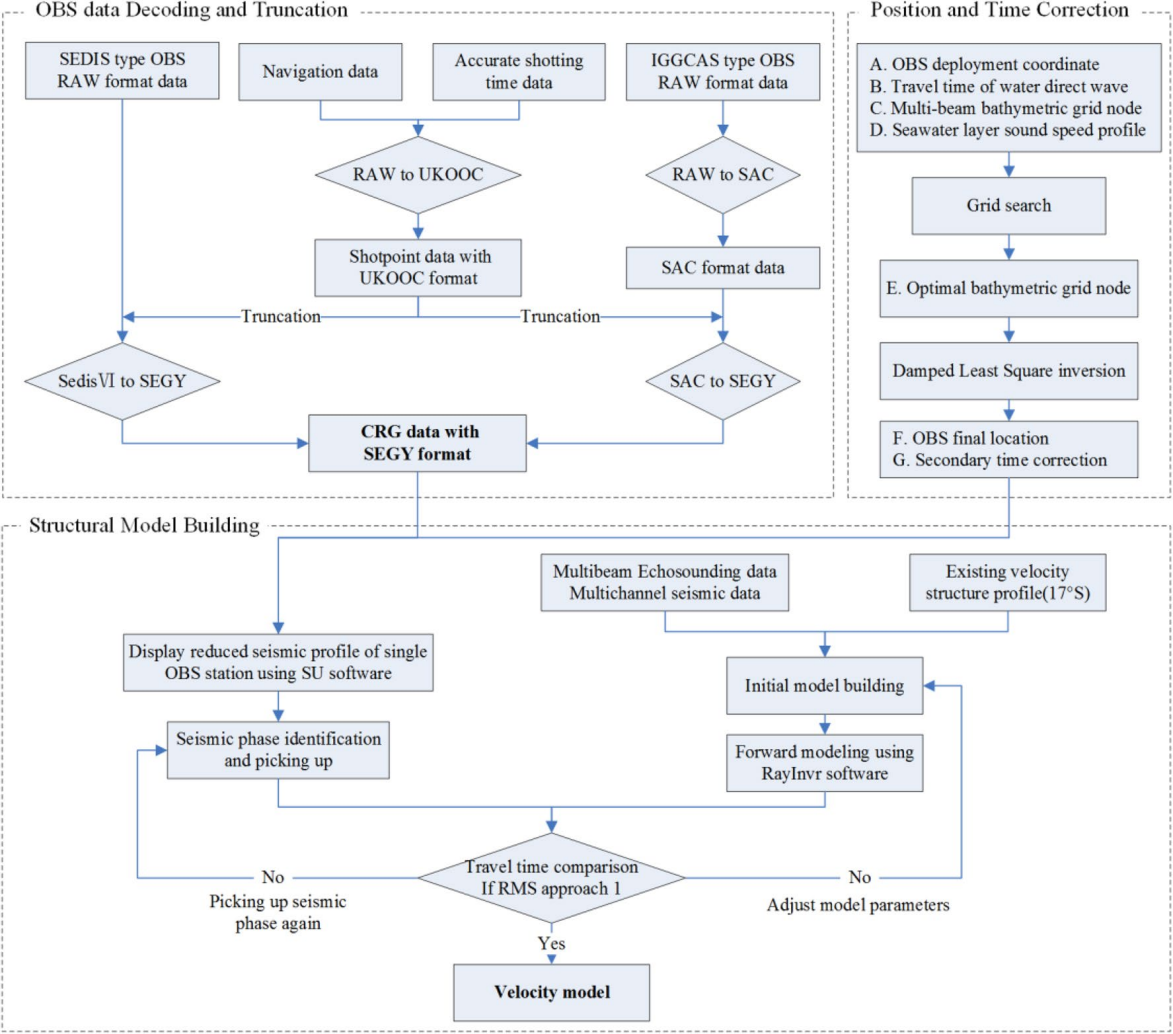
OBS data processing process.

The processing of the longitudinal wave data was determined according to the practice of Chinese scientists conducting active-source OBS surveys in the Bohai Sea and Huanghai Sea^10,18,19^, the East China Sea^20,21^, the South China Sea^22–25^, and the Southwest Indian Ocean^26^ and by referring to the OBS imaging methods employed for the Emperor Seamount Chain and Louisville Ridge in the Pacific Ocean and the Whale Ridge in the Atlantic Ocean^27–29^, as shown in Fig. 11. The workflow primarily included the following aspects:

OBS data decoding and truncation: the precise time and position information of the excitation point is the key for active-source OBS data truncation preprocessing^16,24,26^. In this survey process, the precision gun controller triggered the AIR GUN ARRAY and recorded the precise firing time, and the position of the excitation point was the ship-borne GPS position. Therefore, the ship-borne GPS position had to be corrected to the position of the center of the AIR GUN ARRAY (the two were 40 m apart), and the Ukooa control file required for data truncation was obtained by combining the position information with the accurate time information. Next, for the two different types of raw OBS data, with the OBS drop point treated as the landing position, the data from the Chinese OBS and German OBS devices were decoded and truncated by using format conversion programs to obtain internationally accepted single-station common receiver gather (CRG) data in the SEGY format.

OBS landing position and time corrections: Inshore surface unhooking and free fall dropping are common dropping methods for OBS surveys^30^. Due to marine environmental factors, such as sea breezes and ocean currents, the location of the OBS landing point obviously deviates from the actual drop point. Additionally, the quartz crystal oscillator used for OBS data collection causes clock drift due to factors such as temperature and humidity^30–33^. These drifts in position and time cause large errors in seismic wave travel time pickup^25,31,34^. The search method and the damped least square method were employed to correct the OBS landing point position and time when the direct water wave travel time was used. The specific methods were as follows: ① Pick up the direct water wave travel time information in the SEGY format as the actual arrival time, use the accurate multibeam water depth grid data as the constraint, select the multibeam grid node in the range of 2 km × 2 km with the dropping point as the center, and calculate the theoretical direct water wave arrival time. ② When comparing the actual and theoretical arrival time, select the minimum travel time residual root mean square (RMS) as the optimal depth grid node position of the OBS after the grid search. ③ Use the damped least square method to iterate the inversion until the RMS is the minimum to obtain the final OBS landing position and the secondary time correction amount. After correction, the data quality of most OBS stations was good. The identified direct water wave Pw, sedimentary layer bottom reflection seismic phase PsP, crustal refraction seismic phase Pg, Moho interface reflection seismic phase PmP, and upper mantle top refraction seismic phase Pn were the basis for deep velocity structure modeling.

**Figure 12:**
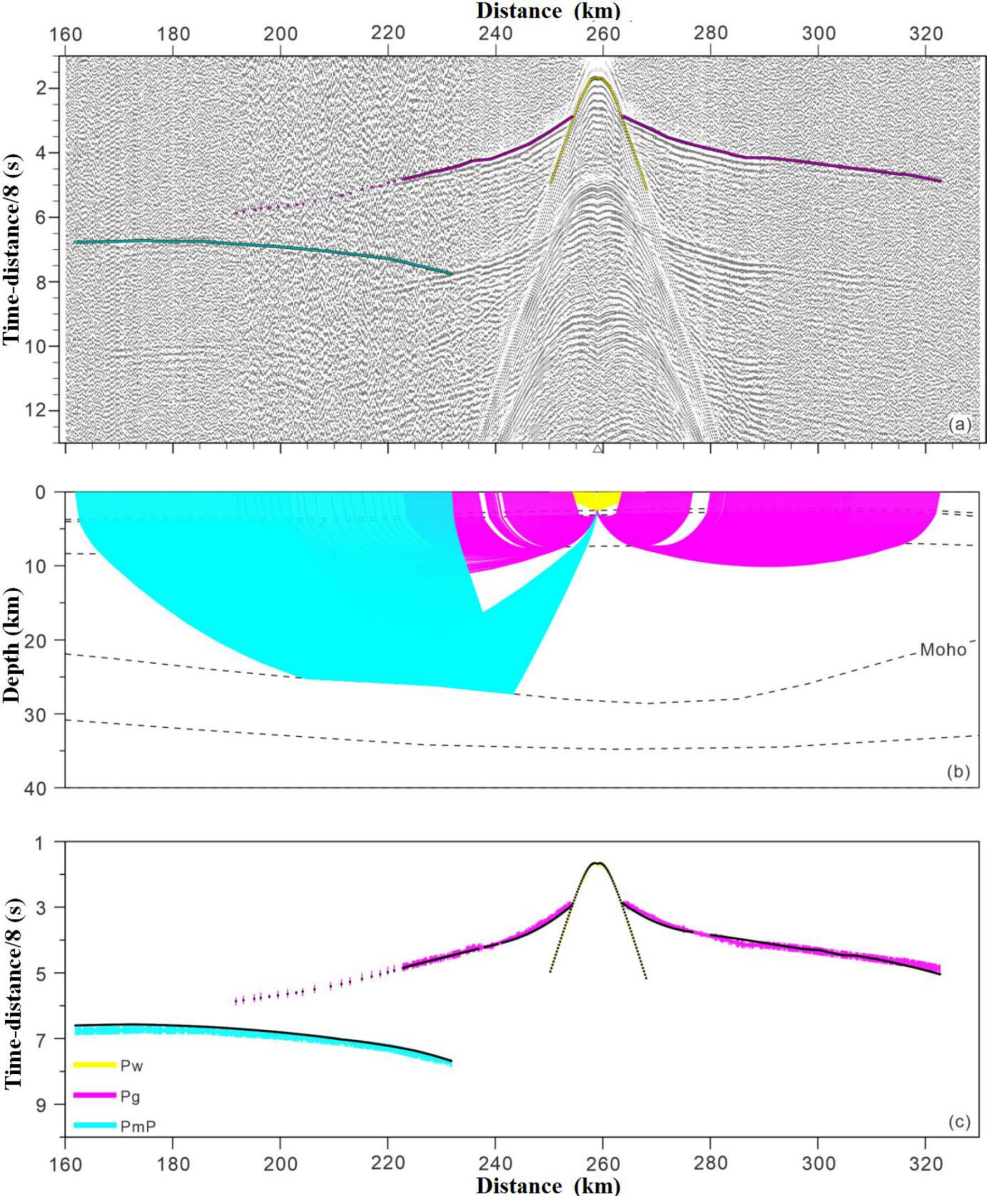
The result of a single station located on the Ninety East Ridge. ((**a**) Seismic record; (**b**) ray tracing; (**c**) travel time fitting results).

Structure model building: when inversion modeling is performed for the ocean active-source OBS wide-angle reflection/refraction seismic travel time, only the refractive seismic phase Pg is used; in addition, the Moho interface reflection seismic phase PmP may be added in some cases. This approach ignores information on fluctuations in the sedimentary basement and has a serious impact on the deep structure simulation results. With the help of the software Rayinvr^35,36^, the best velocity structure model can be obtained by constructing a layered initial model, using the velocity distribution in the refraction wave constrained layer and the fluctuation of the reflection wave constrained interface, and performing repeated forward model fitting by the trial and error method, which has a relatively strong dependence on the initial model. Therefore, an accurate initial model with better control of the shallow strata helps reduce the forward modeling time and quickly obtain the optimal crustal structure model^36^. For this survey, the survey line was arranged to cross the Central Indian Ocean Basin, Ninety East Ridge and the Wharton Basin from west to east. The shallow sedimentary layer had a thickness of 100–800 m, and the sedimentary base fluctuated greatly. Using only the PsP seismic phase would have increased the uncertainty of the shallow stratum results, interfered with the effective fitting of the Pg seismic phase at the near offset distance of the OBS station, and caused the simulation to deviate from the true Moho surface depth and structure of the magma underplating body. Therefore, an initial model was constructed according to the multibeam central beam and MCS data on board, and this model could well restrict the morphology of the seabed and sedimentary substrate and the velocity distribution of the sedimentary layer. Comprehensive analysis was performed on the seismic phase characteristics of different stations along the survey line, and the Rayinvr software package was employed for ray tracing and forward modeling calculations^35^. As a result, the two-dimensional velocity structure model below the survey line was obtained on the top of the Ninety East Ridge, as shown in Fig. 12. This can be compared with the 17°S section on the Ninety East Ridge^37^.

## Conclusion

In this geophysical cross-section survey in the Indian Ocean, a new method was designed and implemented to proportionally excite different seismic sources and synchronously receive signals from the sea surface and seabed. Three sets of seismic profiles with completely different resolutions and penetration depths were obtained through one firing operation, greatly improving the efficiency of deep seismic surveys. Compared with the conventional method, the new method provided in this article can obtain three different resolution stratigraphic profiles through one shot. The G gun seismic profile can provide high-resolution stratigraphic information above the bedrock. Bolt gun seismic profiles can provide lower resolution stratigraphic information above the bedrock and information of Moho surface. And the velocity structure profile of OBS can characterize the crustal structure above the Moho surface.

Unlike traditional seismic profiles, the seismic profiles obtained by this method have a "take-off time" phenomenon due to trigger delays between different air gun, and energy imbalance due to different gas gun capacities. These issues need to be solved before conventional seismic data processing.

Similarly, if the seismic source was replaced by an electric spark seismic source and an air gun seismic source, a shallow high-resolution seismic profile and a seismic profile with a deeper detection depth but a relatively low resolution could be simultaneously obtained.

## Data Availability

The datasets generated and analyzed during the current study are not publicly available because data acquisition was supported by a project from the Ministry of Natural Resources. We do not have permission to share data, but more information is available from the corresponding author on reasonable request.
